# The significance of small lymph nodes on CT for advanced poorly cohesive gastric carcinoma

**DOI:** 10.1186/s40644-026-00991-4

**Published:** 2026-01-21

**Authors:** Gyeongme Cho, Jae Yong Park, Eun Sun Lee, Hyun Ho Shin, Hyun-Wook Park, Hyun Jeong Park, Hee Sung Kim, Jong Won Kim, Beom Jin Kim, Jae Gyu Kim

**Affiliations:** 1 Department of Radiology, Chung-Ang University College of Medicine, Seoul, South Korea; 2 Department of Internal Medicine, Chung-Ang University College of Medicine, Seoul, South Korea; 3 Department of Preventive Medicine, Chung-Ang University College of Medicine, Seoul, South Korea; 4 Department of Pathology, Chung-Ang University College of Medicine, Seoul, South Korea; 5 Department of Surgery, Chung-Ang University College of Medicine, Seoul, South Korea

**Keywords:** Gastric cancer, Lymph nodes, Metastasis, Multidetector computed tomography

## Abstract

**Objective:**

This study aims to assess the size of metastatic lymph nodes (LNs) in advanced poorly cohesive gastric carcinoma (PC-GC) on pre-operative CT and to evaluate potential differences based on histological types.

**Methods:**

We included advanced gastric cancer patients who underwent surgery at Chung-Ang University Hospital from February 2018 to May 2023. Two abdominal radiologists, in consensus, reviewed abdominal CT scans to evaluate the presence of gastric cancer and the largest size of visible LNs at each gastric LN station on CT scans. Measurable LNs were correlated with the full pathology report from electronic database records to determine the metastatic status. We used the independent t-test to evaluate the size difference of metastatic LNs across different histologic types.

**Results:**

We evaluated a total of 136 patients (mean age, 67.25 ± 11.97; 91 males) with advanced gastric cancers. Among them, PC-GC was found in 26 patients (19.1%). Out of 427 measurable LNs, 216 were identified as malignant based on the pathology report. metastatic LNs in PC-GC were significantly smaller than metastatic LNs in other types of gastric cancer (*p* < 0.001, mean ± standard deviation, 6.226 ± 2.538 mm vs. 8.825 ± 5.598 mm). The best cut-off size of the metastatic LNs was 6 mm in PC-GC, as compared with 8 mm in other types of gastric cancers.

**Conclusions:**

The size of metastatic LNs in advanced PC-GCs is significantly smaller than metastatic LNs in other types of gastric cancer. Therefore, lowering the size threshold to 6 mm could enhance pre-operative CT evaluation of metastatic LNs in PC-GC.

**Supplementary Information:**

The online version contains supplementary material available at 10.1186/s40644-026-00991-4.

## Introduction

Gastric cancer is the fifth most common cancer worldwide and the third leading cause of death among all solid cancers according to the 2020 global cancer statistics [1]. Since the 4th edition released in 2010, the WHO classification has included the histological subtype of poorly cohesive carcinoma (PCC), which encompasses signet ring cell carcinoma. This subtype is distinct from other adenocarcinoma subtypes such as tubular, papillary, mucinous and mixed adenocarcinomas. According to alternative Lauren classification, PCCs are classified as ‘diffuse type’ [2]. Specifically, poorly cohesive carcinoma refers to subtypes composed of isolated or small aggregates of poorly cohesive neoplastic cells without gland formation. This distinction is clinically significant as despite the declining overall incidence of gastric cancers worldwide, the incidence of poorly cohesive gastric carcinomas (PC-GCs) is rising in Europe and the United States [3–5]. While the incidence of intestinal-type gastric cancer (GC) has been declining globally compared to the past, the relative proportion or incidence of diffuse-type GC has remained stable or shown an increasing trend in various geographic regions [6–8]. In addition, it is more frequently diagnosed in younger patients (< 45 years), shows a higher prevalence in females, and often presents at a more advanced stage compared to the intestinal type [4, 8, 9].

The prognosis of PC-GC compared with other types of gastric cancers has remained controversial [9, 10]. However, for advanced stage of PC-GC, the prognosis is worse owing to its aggressive histological features, manifesting as higher T-categories (T3 or T4) due to deeper invasion, a greater burden of nodal involvement (N2 or N3), and more frequent peritoneal dissemination (M1) at the time of diagnosis [3, 9, 11–13].

Lymph node (LN) metastasis is a well-known prognostic factor in gastric cancer [14]. Various modalities can be used to evaluate the nodal metastasis in gastric cancer patients, including computed tomography (CT), magnetic resonance imaging, positron emission tomography, endoscopic ultrasound, and even intraoperative sentinel LN biopsy. Among them, abdominal CT is the most frequently used method for preoperative LN metastasis evaluation because of its good accessibility and noninvasiveness, despite variable sensitivity and specificity ranging from 65 to 97% and 49 to 90%, respectively [14–17], primarily based on widely accepted size criteria of 8 mm in short diameter.

However, considering histologic characteristics of isolated or small aggregates of tumor cells and higher rates of LN metastasis in advanced PC-GC, the application of the same size criteria of the “8 mm” for determining metastatic LN could be worth revalidating. Therefore, our aim was to evaluate advanced PC-GCs, with a focus on the difference in size of metastatic LNs compared to other types of gastric cancers in this study. This could potentially enhance the diagnostic performance of preoperative CT scans in identifying metastatic LNs in advanced PC-GCs.

## Materials and methods

### Data collection

In our study, 345 patients who underwent gastric cancer surgery at Chung-Ang University Hospital from February 2018 to May 2023 were analyzed. A total of 136 consecutive advanced gastric cancer (AGC) patients were included in the study. Notably, none of the patients with PC-GC received neoadjuvant chemotherapy prior to surgery. Only one patient with poorly differentiated tubular adenocarcinoma had received only single cycle of chemotherapy three months before surgery.

Advanced gastric cancer was defined, based on the TNM staging of the 8th edition of the American Joint Committee on Cancer (AJCC) staging system, as tumors with T2 stage or higher, regardless of N staging. This is consistent with East Asian clinical practice guidelines distinguishing early (T1) from advanced gastric cancer [2, 18].

**Fig. 1:**
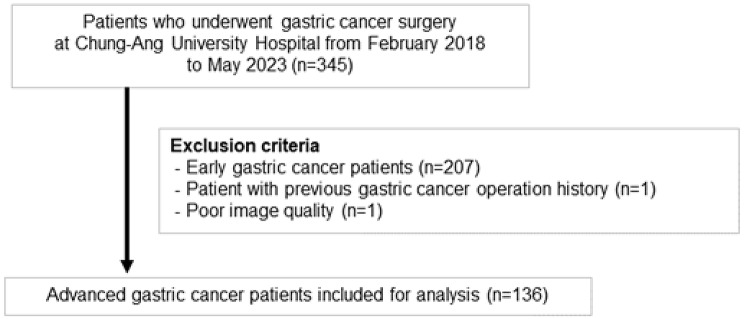
Patient selection

The exclusion criteria were as follows: (1) Patients with early gastric cancer according to the AJCC staging system (*n* = 207), (2) patients who had already undergone a stomach cancer operation (*n* = 1), and (3) those with poor image quality in their scans (*n* = 1) (Fig. 1). Based on pathologic reports from electronic database records, each patient’s TNM staging, histopathological subtype according to the WHO 5th edition, and the stations of surgically removed LNs were recorded retrospectively.

The location of the primary tumor was determined according to the Japanese Classification of Gastric Carcinoma, categorized as upper, middle, lower, or diffuse (involving more than two-thirds of the stomach) [18].

### CT protocol

Out of the total 136 patients, 121 underwent pre-operative CT at our institution, and the analyses included enhance abdomen & pelvis CT for 17 patients, abdomen & pelvis dynamic CT for 20 patients, and standard stomach CT protocols for 84 patients. All CT examinations of our institution were performed using scanners with 64 or more detectors, e.g., Brilliance iCT 256 (Philips), IQon Spectral CT (Philips), Optima CT660 (GE Healthcare). The remaining 15 patients were referred to our institution with pre-operative CT scans already performed at outside hospitals. These scans were obtained using various scanners including Lightspeed 16 (GE Healthcare), Aquilion ONE (Canon), SOMATOM Definition AS (Siemens Healthcare).

Most of the patients underwent the stomach CT protocol for pre-operative analysis at our institution. This CT protocol is widely implemented in South Korea and further optimized at our institution for better evaluation of gastric wall abnormalities [2]. For adequate distension of the stomach, patients were given two packets of effervescent agents (Balgin effervescent granule, 4.0 g; Withhealthcare, Seoul, Korea) and a sip of water before the image acquisition. Non-contrast scans were obtained in the supine position. Then, an intravenous injection of 100–120 mL of iodine contrast agent (the volume variable depending on patient weight) was administered at a flow rate of 3-3.5 mL/sec. The arterial phase scan was obtained in the left posterior oblique position, particularly for optimal distension of the gastric antrum, where gastric cancer most frequently occurs, using the bolus tracking method. The scans were acquired 15 s after the descending thoracic aorta reached 150 HU. Approximately 80 s after the arterial phase scan, portal venous phase scans were acquired in the right decubitus position, particularly for the optimal distension of the gastric fundus.

After CT acquisition, the obtained raw data were transferred to a CT dedicated workstation (Intellispace Portal, Philips, Amsterdam, Netherlands) for multiplanar reformatting, including axial, coronal, sagittal, and surface shaded display imaging. For two-dimensional imaging, the scan interval and thickness were set at 3 mm.

### Analysis of CT images

**Fig. 2:**
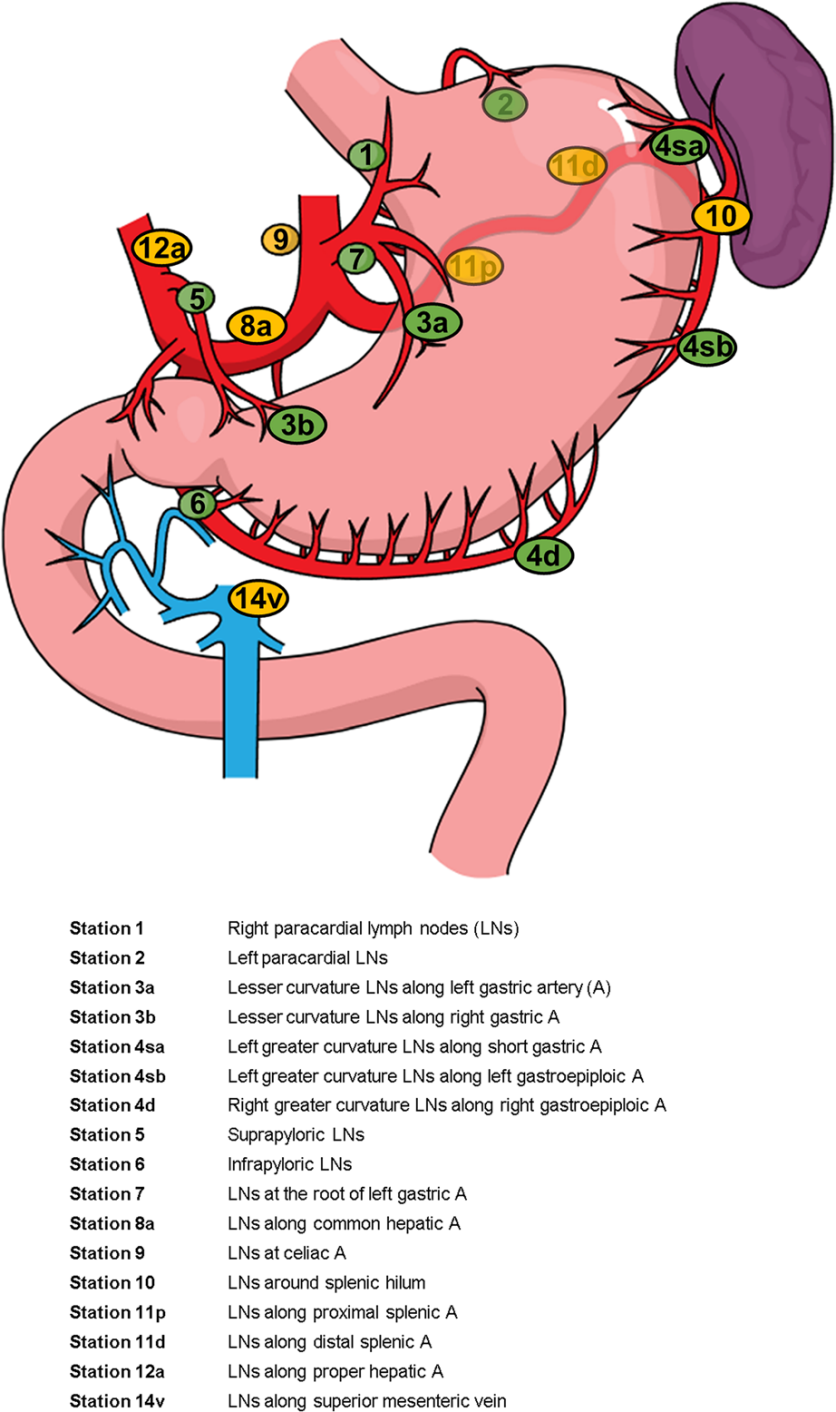
Gastric lymph node station

The pre-operative CT scans of the patients were reviewed in consensus by two abdominal radiologists (Eun Sun Lee and Gyeongme Cho, with 17 and 2 years of experience, respectively). The largest short diameter of measurable LNs at each gastric LN station (Fig. 2) was recorded. Measurable LNs were defined as those with a short diameter of 3 mm or greater, based on 3 mm of slice thickness of CT scanning. Then, detected LNs on pre-operative CT scans were matched with the pathology report in our electronic database system, and the status of metastasis was determined.

### Statistical analysis

The statistical analyses were performed on per-station by using Python software. Student’s *t* test was utilized to evaluate the size difference of metastatic LNs in PC-GC, compared with other types of advanced gastric cancers. The size of LNs was log-transformed due to heavy skewness to the right. For the same reason, statistics using the geometric mean (GM) in addition to the arithmetic mean were also calculated.

The area under the receiver operating characteristic curve (AUROC) was calculated to determine the adequate cut-off for the size of metastatic LNs in PC-GCs. The cut-off value for the size of metastatic LNs was determined by Youden’s J statistic. Furthermore, at the optimal cut-off value, the sensitivity, specificity, accuracy, positive predictive value (PPV), and negative predictive value (NPV) were also calculated.

To identify independent determinants of LN size and to control for the potential confounding effect of T-stage, we performed a linear mixed model (LMM) analysis. Log-transformed lymph node size was used as the dependent variable. Histopathologic type, T-stage, and metastatic status of LNs were included as fixed effects, while patient ID was incorporated as a random effect to account for within-patient clustering. A P value of less than 0.05 was considered statistically significant.

## Results

### Demographic and clinicopathologic characteristics

The mean age of the patients (*n* = 136) was 67.25 ± 11.97 (mean ± standard deviation), and 91 of them were male (66.9%). The histopathologic subtypes of advanced gastric cancers included well-differentiated, moderately differentiated, and poorly differentiated tubular adenocarcinomas (*n* = 8, *n* = 50, and *n* = 35, respectively), poorly cohesive carcinoma (*n* = 26, 19.1%), gastric cancer with lymphoid stroma (*n* = 5), mucinous carcinoma (*n* = 5), neuroendocrine carcinoma (*n* = 2), mixed adeno-neuroendocrine carcinoma (*n* = 2), hepatoid carcinoma (*n* = 1), well-differentiated papillary adenocarcinoma (*n* = 1), and adeno-squamous carcinoma (*n* = 1).

Regarding the tumor location, the lower third of the stomach was the most common site in both groups (*n* = 19 (73.08%), *n* = 61 (55.46%) in the PC-GC and other histopathologic subtypes group, respectively). Specifically, in the PC-GC group, the tumor was located in the mid third in 19.23% (*n* = 5) of cases and in the diffuse or lower third in the remaining cases. Statistical analysis revealed no significant difference in the distribution of tumor locations between the PC-GC group and the other histopathologic subtype (p-value = 0.264).

Out of 136 patients, nodal metastasis based on TNM staging was confirmed in 93 patients (68.4%). Among the 93 patients with nodal metastasis, 21 patients were pathologically proven as poorly cohesive gastric cancer (PC-GC). No significant differences were noted between the patients with PC-GCs and other types of gastric cancers except for pathologic T staging (Table 1).

**Table 1:** Clinicopathologic characteristics in study population (*n* = 137)

	Poorly cohesive carcinoma (*n* = 26)	Other histopathologic subtypes (*n* = 110)	Overall (*n* = 136)	*P*-value
Age	67.42 ± 13.69	67.21 ± 11.59	67.25 ± 11.97	0.935
Sex				0.115
Male	14 (53.85%)	77 (70.00%)	91 (66.91%)	
Female	12 (46.15%)	33 (30.00%)	45 (33.09%)	
Pathologic T staging				0.003
T2	6 (23.08%)	27 (24.55%)	33 (24.26%)	
T3	2 (7.69%)	42 (38.18%)	44 (32.35%)	
T4a	18 (69.23%)	39 (35.45%)	57 (41.91%)	
T4b	0 (0.0%)	2 (1.82%)	2 (1.47%)	
Pathologic N staging				0.057
0	5 (19.23%)	38 (34.55%)	43 (31.62%)	
1	2 (7.69%)	24 (21.82%)	26 (19.12%)	
2	9 (34.62%)	19 (17.27%)	28 (20.59%)	
3a	4 (15.38%)	17 (15.45%)	21 (15.44%)	
3b	6 (23.08%)	12 (10.91%)	18 (13.24%)	
Pathologic M staging				1.000
0	23 (88.46%)	98 (89.09%)	121 (88.97%)	
1	3 (11.54%)	12 (10.91%)	15 (11.03%)	
Tumor location				
Upper third	0 (0.0%)	10 (9.09%)	10 (7.35%)	0.264
Mid third	5 (19.23%)	31 (28.18%)	36 (26.47%)	
Lower third	19 (73.08%)	61 (55.46%)	80 (58.83%)	
Diffuse	2 (7.69%)	8 (7.27%)	10 (7.35%)	

Note. Continuous data (age) presented as mean ± standard deviation and categorical variables are presented as number of patients, with percentages in parentheses. *P* values were calculated using the Student’s *t* test for continuous variables, Chi-square test for categorical variables and Fisher’s exact test for Pathologic T staging and tumor location

Regarding the distribution of pathologically proven metastatic lymph nodes (LNs), station 3 exhibited the highest frequency of metastasis, accounting for 24.07% (*n* = 52) of the total metastatic LNs. This was followed by station 4d and station 6, which showed 14.35% (*n* = 31) and 13.89% (*n* = 30), respectively.

Significant size differences and optimal cut-off values for metastatic LN size in PC-GC.

Total 427 LNs were measured (short diameter ≥ 3 mm) on pre-operative CT scans of the enrolled patients. Out of them, 216 LNs (50.6%) were matched as metastatic LNs based on pathology report. Among the 216 metastatic LNs, 62 LNs (28.7%) were present in PC-GC. Based on Student’s *t* test, the size of metastatic LNs in PC-GC was significantly smaller than metastatic LNs in other types of gastric cancer (*p* < 0.001, arithmetic mean ± standard deviation (SD), 6.226 ± 2.538 mm vs. 8.825 ± 5.598 mm; geometric mean (geometric standard deviation, GSD): 5.774 mm (1.472) vs. 7.613 mm (1.683)) (Table 2).

**Table 2:** Clinical and pathologic findings of surgically resected lymph nodes

	Poorly cohesive carcinoma	Other histopathologic subtypes	Overall	*P*-value
Measurable Lymph nodes				
Number	91	336	427	
Size on CT, Mean ± SD (mm)	5.736 ± 2.375	7.185 ± 4.361	6.876 ± 4.062	< 0.001
Size on CT, GM (GSD) (mm)	5.325 (1.460)	6.368 (1.582)	6.130 (1.566)	< 0.001
Metastatic lymph nodes (*n* = 216)				
Number	62	154	216	
Size on CT, Mean ± SD (mm)	6.226 ± 2.538	8.825 ± 5.598	8.079 ± 5.051	< 0.001
Size on CT, GM (GSD) (mm)	5.774 (1.472)	7.613 (1.683)	7.032 (1.650)	< 0.001
Non-metastatic lymph nodes (*n* = 21**1**)				
Number	29	182	211	
Size on CT, Mean ± SD (mm)	4.690 ± 1.561	5.797 ± 2.120	5.645 ± 2.084	0.008
Size on CT, GM (GSD) (mm)	4.479 (1.350)	5.475 (1.393)	5.326 (1.397)	0.002

Note. Lymph node size measured as short diameter of the lymph nodes, represented as mean ± standard deviation

CT, computed tomography; SD, standard deviation; GM, geometric mean; GSD, geometric standard deviation

**Fig. 3:**
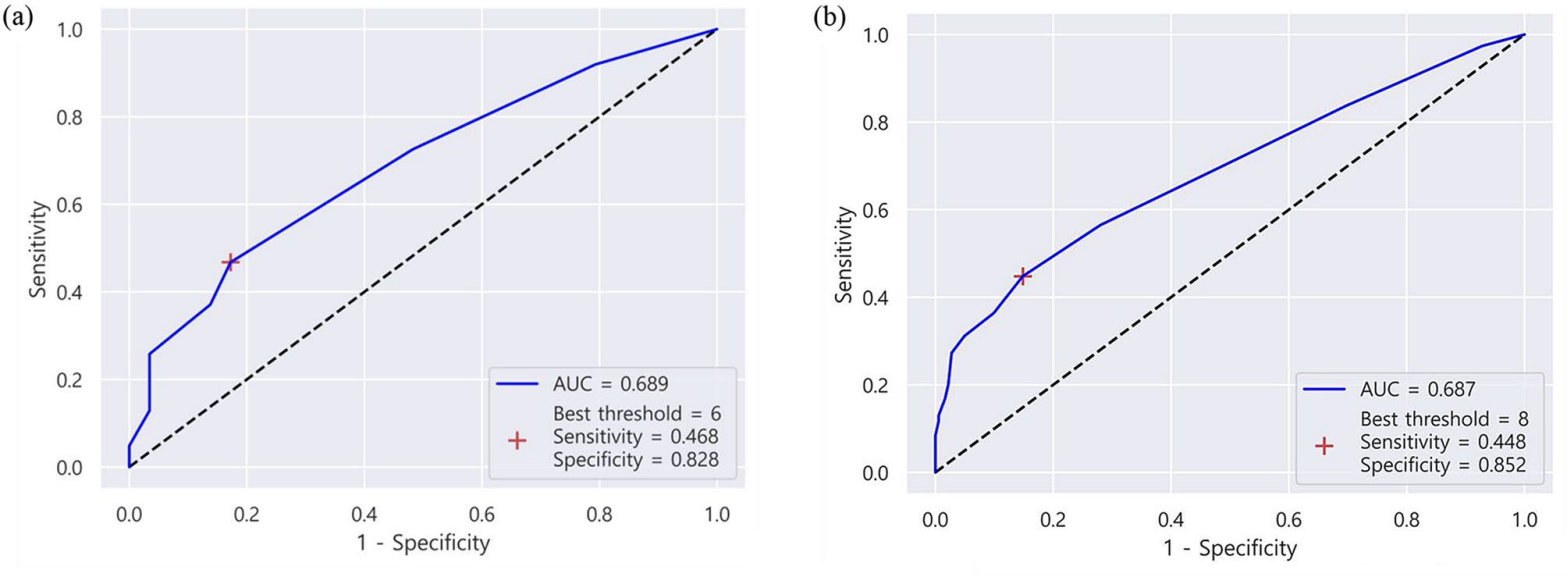
Receiver operating characteristics curve for metastatic lymph nodes in poorly cohesive carcinomas (**a**) and other histopathologic subtypes (**b**)

The best cut-off size of the metastatic LNs was 6 mm in PC-GC, which had an AUROC of 0.689 (95% CI: 0.578, 0.800) (Fig. 3A). Furthermore, the sensitivity, specificity, PPV, and NPV were 0.468, 0.828, 0.853, and 0.421, respectively. In other types of gastric cancer, the optimal size cut-off of the metastatic LNs was 8 mm and its AUROC was 0.687 (95% CI: 0.629, 0.744) (Fig. 3B). The sensitivity, specificity, PPV and NPV were 0.448, 0.852, 0.719, and 0.646, respectively.

### Determinants of LN size

In the LMM analysis (Table 3), the poorly cohesive histologic type was significantly associated with smaller lymph node size compared with other subtypes (β = −0.187 [95% CI: −0.338, − 0.035], p-value = 0.016). In contrast, T-stage was not statistically associated with lymph node size (T3 vs. T2: β = 0.132 [95% CI: −0.032, 0.295], p-value = 0.116; T4 vs. T2: β = 0.088 [95% CI: −0.066, 0.243], p-value = 0.263).

**Table 3:** Linear mixed model analysis for factors associated with lymph node size

	Coefficient (95% CI)	*P*-value
Histopathologic Type		
Other subtypes	Reference	
Poorly cohesive	-0.187 (-0.338, -0.035)	0.016
T-stage		
T2	Reference	
T3	0.132 (-0.032, 0.295)	0.116
T4	0.088 (-0.066, 0.243)	0.263
Metastasis		
Non-Metastatic	Reference	
Metastatic	0.258 (0.178, 0.338)	< 0.001

Note: Categorical variables are presented as numbers with percentages in parentheses. P values were calculated using a linear mixed model (LMM) to account for within-patient clustering, treating patient ID as a random effect. LN size was log-transformed before analysis to achieve a normal distribution

## Discussion

Previous studies have highlighted the importance of pre-operative CT evaluation for nodal metastasis [19–23]. Specifically, key CT characteristics associated with metastatic lymph nodes include a round shape, central necrosis, and heterogeneous enhancement. Furthermore, lymph nodes measuring more than 1 cm without a fatty hilum, exhibiting marked enhancement (over 80 or 100 HU), or appearing in clusters of more than three are commonly regarded as indicators of malignancy [24, 25]. Out of the various characteristics, size has emerged as the most reliable measure for determining nodal metastasis, with a worldwide consensus [26, 27].

**Fig. 4:**
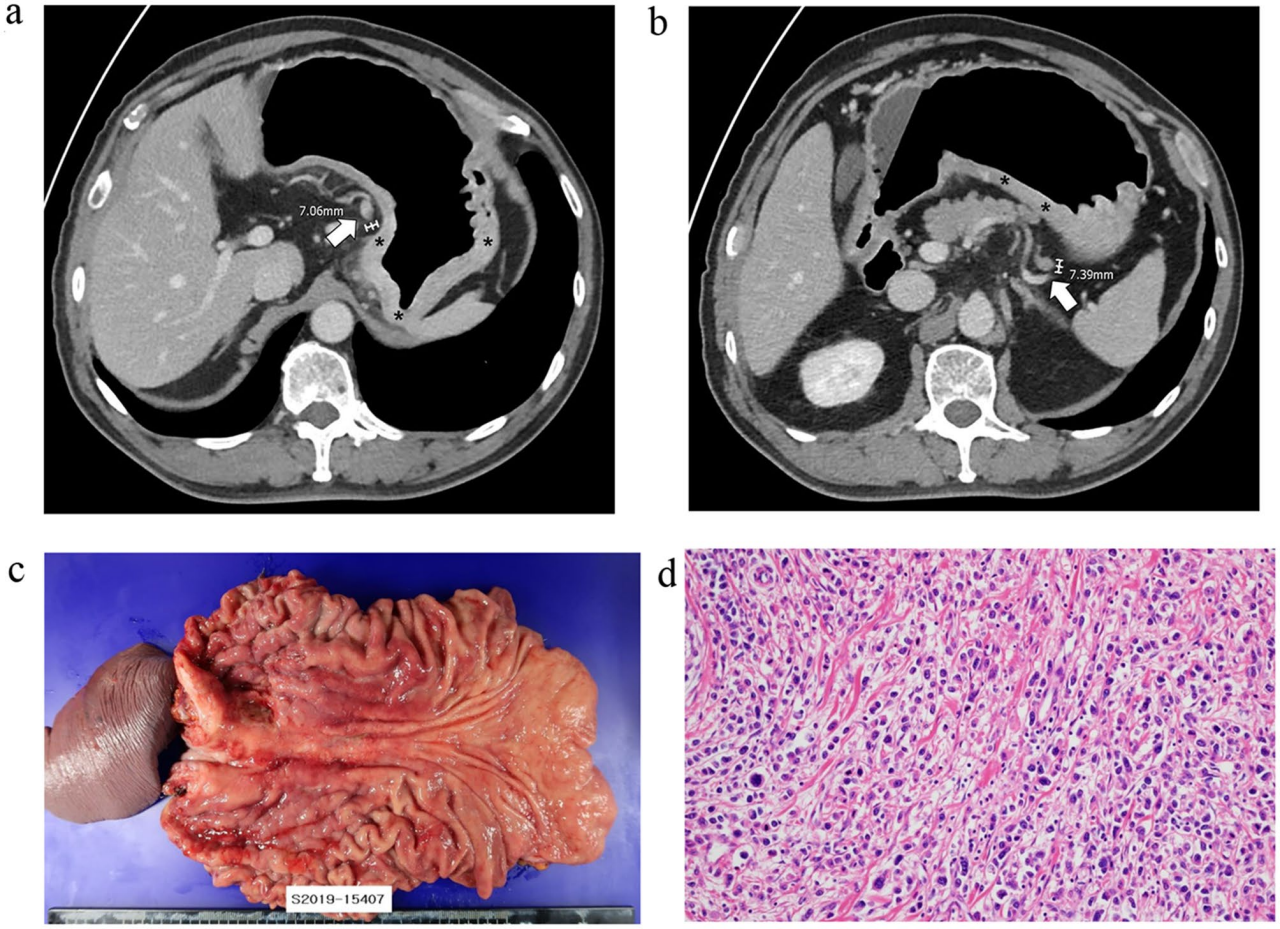
A 57 year-old male patient diagnosed with localized Borrmann type IV advanced gastric cancer. Several lymph nodes measuring up to 7 mm were found along the lesser curvature and along the proximal splenic artery (**a**, **b**). Surgical specimen revealed poorly cohesive carcinoma with serosa invasion, and CT detected lymph nodes were confirmed to be metastatic (**c**). On the pathological slide, isolated tumor cells without definite gland formation were observed (**d**)

**Fig. 5:**
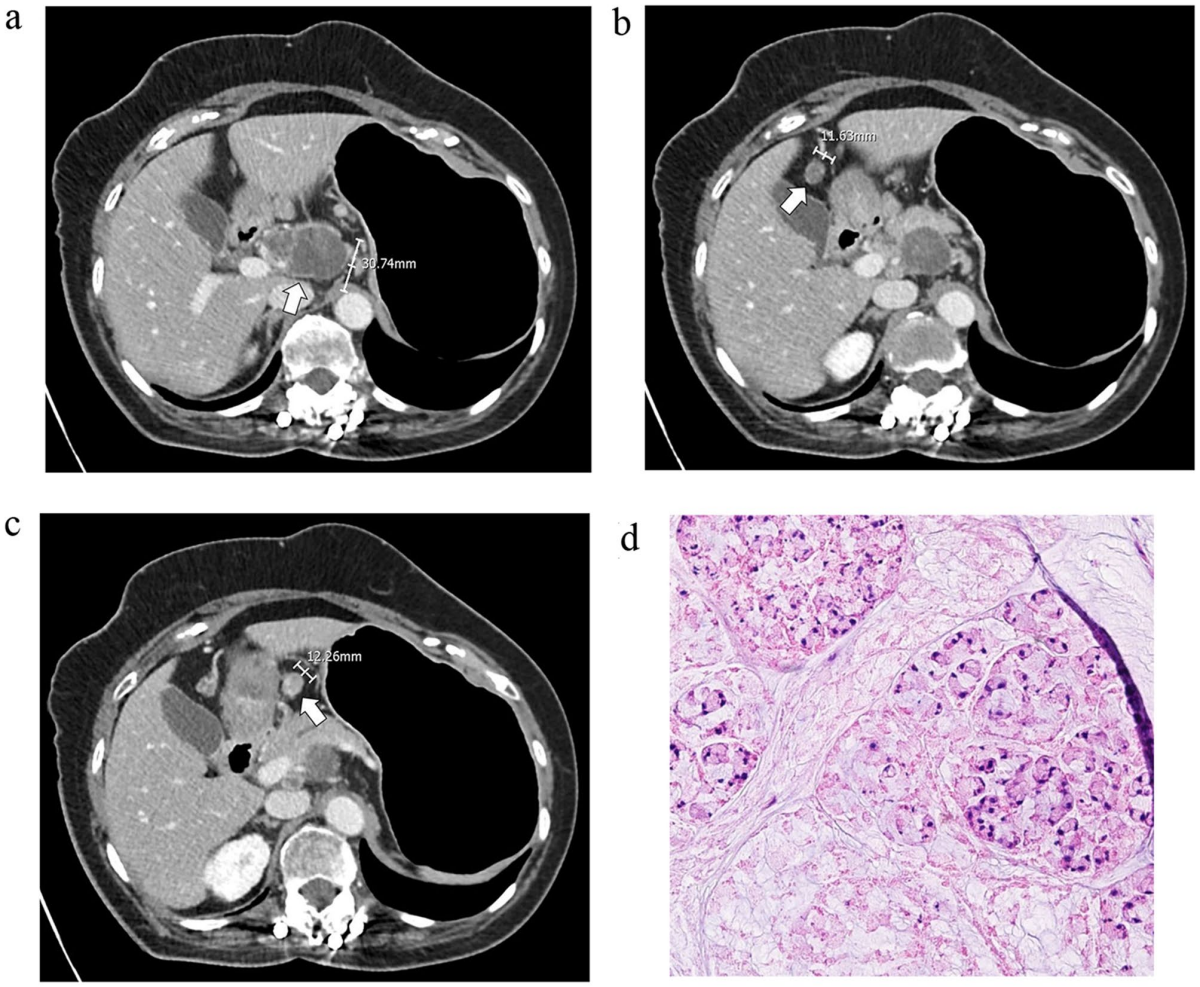
A 65 year-old female patient diagnosed with advanced gastric cancer. Preoperative CT imaging revealed lymph nodes measuring up to 30 mm in the common hepatic station, as well as 12 mm and 11 mm lymph nodes in the supra- and infrapyloric stations, respectively (**a**-**c**). Pathological examination confirmed these lymph nodes to be metastatic mucinous carcinoma (**d**)

Notably, this study is the first to assess the size of metastatic LNs in advanced PC-GC. We have demonstrated that the metastatic LNs in PC-GC are significantly smaller than those in other types of gastric cancer (*p* < 0.001, GM (GSD): 5.774 (1.472) vs. 7.613 (1.683)). Furthermore, we determined the optimal cut-off size for metastatic LNs in PC-GC to be 6 mm (AUROC: 0.689, 95% CI: 0.578-0.800), compared to 8 mm in gastric cancers with other histologic types (Figs. 4 and 5). This finding inherently leads to the necessity of lowering the size threshold for metastatic LNs in PC-GCs.

A critical strength of this finding lies in its robustness against potential confounding by tumor stage. It is noteworthy that the PC-GC group in our study had a significantly higher proportion of advanced T-stage tumors, particularly T4a, compared to other histopathologic types. Theoretically, advanced T-stage contributes to lymph node enlargement, a trend also suggested by our LMM analysis. Nevertheless, despite this high burden of LN enlargement, LNs in the PC-GC group remained significantly smaller in LMM analysis.

Until now, many studies have adopted an 8 mm size threshold for metastatic LNs in gastric cancers on pre-operative CT scans [25]. However, the diagnostic performance of the CT scan has been somewhat underwhelming, with specificity ranging widely from 62.5% to 91.9% and sensitivity varying between 50% and 87.9% across various studies [24, 25]. Considering the results of our study, employing a lower threshold when evaluating pre-operative CT scans for biopsy-proven PC-GC could significantly improve the diagnostic ability of these scans.

Regarding the surgical approach to gastric cancers, D2 lymphadenectomy is the routinely performed standard in Japan and South Korea, where the incidence of gastric cancer is high. However, less extensive D1 lymphadenectomies are often performed in countries with a lower incidence, such as the United States [28, 29]. Being aware of the significance of small LNs in PC-GC naturally prompts surgeons to exercise extra caution. In such cases, D2 lymphadenectomy might be considered over D1 lymphadenectomy as the standard practice, ensuring the thorough harvesting of even small LNs that may harbor malignant potential.

This study employs a distinct approach to assess metastatic LNs in AGCs, yielding unique results compared to previous studies. As mentioned earlier, there have been studies investigating various CT features of metastatic LNs in gastric cancers, including shape, necrosis, enhancement pattern, and clustering [26, 27, 30]. However, we did not evaluate these characteristics in this study for the following reasons. First, the majority of the LNs included in our study are smaller than 1 cm, which could result in lower reliability and reduced objectivity when assessing these characteristics on pre-operative CT scans by individual radiologists (mean size of the LNs, 6.876 ± 4.062 mm (mean ± SD)). Second, we chose the measurable LNs (≥ 3 mm) with the largest short diameter to represent each gastric LN station, indicating that the representative LN does not reflect the various characteristics of all the LNs in the corresponding station. Therefore, we only evaluated the most objective size criterion and were able to obtain significant results. Nevertheless, further studies considering the various characteristics of metastatic LNs in PC-GC could yield results with increased significance, though many practical obstacles exist.

Endoscopic resection is currently the treatment of choice for early gastric cancers with negligible risk of LN metastasis due to its less invasive nature and oncologic outcomes comparable to surgery [31, 32]. Recent studies even suggest that endoscopic submucosal dissection (ESD) could be chosen as an optimal treatment for PC-GC that meet the expanded indications outlined in the guidelines of the Japanese Gastric Cancer Association (JGCA) [33, 34]. However, our study’s highlighting of the significance of small-sized LNs in PC-GC calls for a reconsideration of this perspective. However, before deciding on ESD, the current standard clinical practice is to first exclude the possibility of LN metastasis through imaging tests such as CT scans. Following recommendations from the aforementioned guidelines, it is expected that ESD will be more widely performed for undifferentiated type EGCs, including PC-GC, in the future. In consideration of this, our study’s emphasis on the significance of small-sized LNs in PC-GC highlights a crucial point in clarifying the criteria for excluding LN involvement in PC-GC using CT scans, potentially influencing the treatment strategy.

The precise measurement of lymph node size on CT can be influenced by technical factors, including slice thickness, reconstruction intervals, and potential motion artifacts. Given that the proposed threshold difference is relatively small (6 mm vs. 8 mm), measurement variability could affect clinical interpretation. Thinner slice acquisitions (e.g., thin-section reconstructions) would theoretically reduce partial volume effects and enhance measurement reliability. Therefore, the application of standardized high-resolution CT protocols is recommended to optimize the diagnostic performance of the 6 mm cutoff in clinical practice.

Our study has several limitations. First, as a retrospective single-center study, the sample size was relatively small. Second, significant differences in pathologic T-staging between the cohorts may have introduced selection bias. In addition, the heterogeneity of the ‘other histopathologic subtypes’ group, which comprises tumors with potentially differing lymph node characteristics, is another limitation. Although robust statistical comparisons among these subtypes were limited by small sample sizes, descriptive data on lymph node sizes for each subtype are provided in Supplementary Table 1 for reference. Therefore, large-scale multi-center studies are needed to validate our findings. Fourth, the measurable LNs (≥ 3 mm in short diameter) were matched to the pathologic report retrospectively, indicating that accurate matching of the dissected LNs was not possible. Furthermore, the surgeon subjectively categorized the harvested gastric LNs by each peri-gastric LN station, further complicating the matching process. Fifth, as mentioned previously, several other characteristics including shape, necrosis, enhancement pattern, and clustering of LNs have been proven to play a role in determining the metastatic status of LNs on pre-operative CT scans [24, 30]. Such characteristics were not considered in this study, indicating the necessity for additional research.

In conclusion, the size of metastatic LNs in advanced PC-GCs is significantly smaller than metastatic LNs in other types of gastric cancer. Therefore, lowering the size threshold to 6 mm could enhance pre-operative CT evaluation of metastatic LNs in PC-GC.

## Electronic supplementary material

Below is the link to the electronic supplementary material.

Supplementary Material 1

## Data Availability

The datasets during and/or analyzed during the current study are available from the corresponding author on reasonable request.
