# Supplementary material for: The significance of small lymph nodes on CT for advanced poorly cohesive gastric carcinoma
Source: Cancer Imaging. 2026 Jan 21;26:27. doi: 10.1186/s40644-026-00991-4 (PMC12905975; doi:10.1186/s40644-026-00991-4)
Supplement: Supplementary file 1 — Supplementary Material 1 [file 40644_2026_991_MOESM1_ESM.docx]

**Supplementary Table 1. Descriptive statistics of lymph node sizes according to histologic subtypes**

|  | Number | Size on CT | |
| --- | --- | --- | --- |
|  |  | Mean±SD (mm) | GM (GSD) (mm) |
| Measurable Lymph nodes | 427 | 6.876±4.062 | 6.130(1.566) |
| Poorly cohesive carcinoma | 91 | 5.736±2.375 | 5.325(1.460) |
| Other histopathologic subtypes | 336 | 7.185±4.361 | 6.368(1.582) |
| WD | 19 | 7.579±3.761 | 6.890(1.537) |
| MD | 127 | 6.488±4.507 | 5.783(1.531) |
| PD | 125 | 7.936±4.006 | 7.121(1.576) |
| Mucinous | 23 | 7.870±5.826 | 6.688(1.703) |
| NEC | 6 | 8.833±4.834 | 7.856(1.690) |
| MANEC | 4 | 12.000±8.485 | 9.898(2.078) |
| Hepatoid | 3 | 6.333±3.215 | 5.848(1.613) |
| Others | 29 | 5.276±1.944 | 4.983(1.399) |
| Metastatic Lymph nodes | 216 | 8.079±5.051 | 7.032(1.650) |
| Poorly cohesive carcinoma | 62 | 6.226±2.538 | 5.774(1.472) |
| Other histopathologic subtypes | 154 | 8.825±5.598 | 7.613(1.683) |
| WD | 6 | 9.833±3.656 | 9.308(1.433) |
| MD | 51 | 7.686±6.635 | 6.358(1.730) |
| PD | 72 | 9.125±4.547 | 8.128(1.618) |
| Mucinous | 12 | 10.667±6.985 | 9.257(1.684) |
| NEC | 4 | 10.750±4.856 | 9.948(1.579) |
| MANEC | 2 | 17.000±9.899 | 15.492(1.857) |
| Hepatoid | 1 | 4.000 | 4.000 |
| Others | 6 | 7.000±2.898 | 6.523(1.507) |
| Non-Metastatic Lymph nodes | 211 | 5.645±2.084 | 5.326(1.397) |
| Poorly cohesive carcinoma | 29 | 4.690±1.561 | 4.479(1.350) |
| Other histopathologic subtypes | 182 | 5.797±2.120 | 5.475(1.393) |
| WD | 13 | 6.538±3.455 | 5.996(1.487) |
| MD | 76 | 5.684±1.775 | 5.427(1.360) |
| PD | 53 | 6.321±2.327 | 5.949(1.416) |
| Mucinous | 11 | 4.818±1.168 | 4.691(1.276) |
| NEC | 2 | 5.000±1.414 | 4.899(1.332) |
| MANEC | 2 | 7.000±4.243 | 6.325(1.912) |
| Hepatoid | 2 | 7.500±3.536 | 7.071(1.633) |
| Others | 23 | 4.826±1.370 | 4.645(1.327) |

Note. Lymph node size measured as short diameter of the lymph nodes, represented as mean ± standard deviation.

CT, computed tomography; SD, standard deviation; GM, geometric mean; GSD, geometric standard deviation
